# Evaluating community engagement efforts in a clinical and translational research initiative

**DOI:** 10.1017/cts.2025.10069

**Published:** 2025-06-09

**Authors:** Kelly Finck Waters, Brenda Joly, Carolyn E. Gray, Jan K. Carney, Kathleen M. Fairfield

**Affiliations:** 1 Muskie School of Public Service, University of Southern Maine (USM), Portland, ME, USA; 2 Larner College of Medicine, University of Vermont (UVM), Burlington, VT, USA; 3 Maine Health Institute for Research, Scarborough, ME, USA; 4 Department of Medicine, Maine Medical Center, Portland, ME, USA

**Keywords:** Collaboration, community-engaged research, social network analysis, evaluation innovation

## Abstract

A priority of the Northern New England Clinical and Translational Research (NNE-CTR) Network is conducting, promoting, and advancing community-engaged research through its Community Engagement and Outreach (CEO) Core. We sought to measure the CEO Core’s success in strengthening community-level research partnerships using a validated survey platform based on network science to map and track collaborations over time. The survey was completed by 59/76 organizations (77.6% response rate). Key findings included a high level of trust and a modest level of perceived value relative to published benchmarks. Additional specific findings will inform opportunities to improve the network as the NNE-CTR matures.

## Introduction

As part of the National Institute for General Medical Sciences’ Clinical and Translational Research Network (CTR-N) Award program, the Northern New England Clinical and Translational Research Network (NNE-CTR) is a multi-state regional initiative that engages academic institutions, healthcare organizations, public health agencies, and local community-based organizations and stakeholders. The NNE-CTR’s Community Engagement and Outreach (CEO) Core serves as the bridge connecting a wide network of partners with the goal of fostering participatory research opportunities that support ongoing bi-directional engagement between communities and investigators.

Efforts to evaluate community-level research partnerships in other clinical and translational research networks have used several approaches including publication data, administrative data, survey data, and network science. For example, some initiatives have used co-authorship analysis to measure collaboration within research networks [1,2] or examined community-engaged research activities based on attendance data from a collaborative research project[3]. Social network analysis and mapping have also been used in combination with bibliometric analyses [3,4]. Additionally, research networks have created surveys specific to their community-engaged efforts to measure collaboration [5]. Other published scales, surveys, and tools exist to measure collaboration among health coalitions and community partnerships; however, reviews have noted a need for reported validity and reliability for many of these tools [6,7].

Expanding on previous efforts, the NNE-CTR’s evaluation team and CEO Core decided to conduct a social network analysis of the NNE-Community Engaged Research Network. This type of analysis was selected to capture and visualize the relationships within the network. It provides a helpful understanding of how the network functions, as well as highlighting strong relationships and key players. Social network analysis is also valuable in uncovering areas where connections are missing or could be enhanced. These insights are especially useful to an expanding network, like the NNE-Community Engaged Research Network, and will help guide the CEO Core’s engagement efforts in the coming years.

The resource used for the NNE-Community Engaged Research Network’s social analysis was the PARTNER (Platform to Analyze, Record, and Track Networks to Enhance Relationships) CPRM (Community Partner Relationship Management) Platform. This resource was selected because it includes a validated survey based on network science, as well as analytic and mapping features, to track collaborations over time [8,9]. A key feature of the survey is the computation of network trust and value scores, along with user-friendly survey tools and mapping software to visualize network characteristics. It has been used since 2008 to analyze community-level partnerships in many different sectors [10–16], and it is a potentially useful tool for evaluating characteristics of community-engaged CTR initiatives over time to assess changes in the strength, activity, and perceived levels of trust and value among participants.

The PARTNER CPRM platform has the ability to conduct a thorough social network analysis while also incorporating more traditional survey questions. The evaluation team’s aim for using this tool is to support its evaluation of how well the CEO Core is strengthening community-level research partnerships in years 1, 3, and 5 of its grant cycle. To gauge this progress, 4 overarching questions are asked:

1. What are the characteristics of community partners engaged in the NNE-Community Engaged Research Network?
2. What are the activities of the NNE-Community Engaged Research Network?
3. How do community partners engage with and view NNE-CTR services?
4. What relationships exist within the NNE-Community Engaged Research Network?

As the survey is repeated in future years, the PARTNER tool will enable the NNE-CTR evaluation team and CEO Core to visualize and track how the NNE-Community Engaged Research Network’s members develop relationships, shift in characteristics, engage in activities, and collaborate with NNE-CTR services.

## Methods

### PARTNER survey

The PARTNER CPRM platform contains the standard PARTNER survey with 19 questions. The survey includes 8 validated items measuring trust and value, and 11 modifiable questions focused on the characteristics of members and the network, as well as members’ perceptions of the network’s goals and progress. PARTNER survey templates are available for different types of networks and include notes about allowable modifications, as well as optional additional questions [17], so that networks can customize the items based on their research or evaluation questions.

The evaluation team worked in collaboration with the CEO Core to revise the modifiable survey items. (Note that the trust and value survey items were not altered in order to maintain their validity.) For example, many of the sample questions were in a “Check all that apply” format. However, the evaluation team omitted this response option format in favor of matrix questions. The team wanted to be able to distinguish between items not checked because they were missing or because the response was no. Other changes to the sample survey items included adjusting the wording of the questions that focused on the characteristics of members and the network (and their response options) so they were more appropriate for a network focused on community engaged research.

The final NNE-Community Engaged Research Network PARTNER survey had 26 items, including the 8 validated relational questions about trust and value, 4 questions about general strategies to facilitate community engagement, 2 items with open-ended responses, 4 questions about organizational characteristics, and 8 items assessing network opinions and engagement.

### Participants

To determine who was eligible for the survey, the CEO Core drafted a list of existing and potential members of the NNE-Community Engaged Research Network. In developing this list, the CEO Core used two public health frameworks [18,19]. Using these broader organizing principles served as a starting point and aspirational framework for network growth and expansion. Members represented various academic/research institutions, community-based organizations, health associations, healthcare organizations, and state or local government entities. In all, 87 members were based in Vermont and 69 members in Maine. An additional 3 members were in New Hampshire, and 1 was from Massachusetts.

Of the 160 total members, the survey was only sent to actively engaged members who had a history of collaboration with the CEO Core, rather than new partners. While selecting a subsample of the larger network population introduced a selection bias, the CEO Core decided it was important to consider their relationships with organizations. It was preferable for the team to hold off on administering surveys to organizations with whom they were still getting to know.

Ultimately, the CEO Core selected 76 members (29 from Maine, 46 from Vermont, and 1 from New Hampshire) who would receive the survey. However, all 160 total members were listed as response options for the relational questions about organizations with whom respondents have an established relationship.

### Administration

The survey was sent via email through the PARTNER platform in April 2023. It remained open for 4 weeks. To increase the response rate, NNE-CTR leadership and CEO Core staff conducted personal outreach to potential respondents via email. Some of these follow-up messages included updates about the response rate and early, anonymized social network maps to generate interest.

## Results

Of the 76 organizations invited to participate, 59 took part in the survey for a response rate of 77.6%. As shown in Table 1, the majority of respondents (61%) have been involved in the NNE-Community Engaged Research Network for more than one year. Every respondent agreed they are motivated to be involved in the network because of a desire to collaborate to address health problems.

**Table 1.** Northern New England Community Engaged Research Network Respondents (*n* = 59)

Sector (*n* = 59)	
Healthcare organization	24 (41%)
Academic/research institution	16 (27%)
Health association	8 (14%)
Community-based organization	5 (8%)
State/local government agency/entity	4 (7%)
Other: business, media/news	2 (3%)
**Time engaged with network** (*n* = 59)	
> 1 year	36 (61%)
6-12 months	8 (14%)
< 6 months	2 (3%)
Other	6 (10%)
Not yet engaged	7 (12%)
Motivation for involvement in the network* (*n* = 59)	
Desire to collaborate to address health problems	59 (100%)
Desire to implement evidence-based practices	58 (98%)
Desire to improve health outcomes	57 (96%)
Desire to improve health equity	57 (96%)
Desire to translate research findings to inform community health access	57 (96%)
Desire to improve health access	56 (94%)
Desire to contribute to research	55 (92%)
Desire to gain knowledge and skills	55 (92%)
Desire to advocate for the community	50 (84%)
Other	14 (23%)
Top 5 areas of research to advance* (*n* = 50)	
Rural health	32 (64%)
Health equity	30 (60%)
Social determinants of health	29 (58%)
Access to healthcare	25 (50%)
Mental health	16 (32%)
Resources available to contribute to the network* (*n* = 49)	
Connections to the community	29 (59%)
Community expertise/knowledge	26 (53%)
Access to potential research participants	25 (51%)
Leadership expertise	25 (51%)
Research expertise	22 (45%)
Content expertise	20 (41%)
Data or data infrastructure	19 (39%)
Clinical expertise	18 (37%)
Facilitation expertise	17 (35%)
Advocacy expertise	16 (33%)
Communication resources (e.g., Listservs)	16 (33%)
Project management expertise	15 (31%)
Training expertise	15 (31%)
Physical resources (e.g., study site)	13 (27%)
Fiscal resources	4 (8%)
Other	4 (8%)
Not sure	3 (6%)

* *Respondents could choose more than one response for these survey items*.

The top 5 areas of research that respondents hope to advance through the NNE-Community Engaged Research Network were rural health, health equity, social determinants of health, access to healthcare, and mental health. When asked which resources they have or could contribute to the network, over half of respondents reported being able to contribute connections to their community (59%), knowledge of their community (53%), access to potential research participants (51%), and leadership expertise (51%).

Respondents’ experiences with both the NNE-Community Engaged Research Network and the NNE-CTR are summarized in Table 2. In terms of how network members have engaged with the network, 86% have participated in meetings, 71% have connected other community partners to the group, and 67% of respondents have collaborated on a research project through the network. Overall, 89% of respondents agreed or strongly agreed that the NNE-Community Engaged Research Network increases opportunities for rural health research. When we queried how members of the NNE-Community Engaged Research Network use NNE-CTR services, over half of respondents reported using community engagement research navigation services (62%) and professional development services (53%). Only 13% of respondents reported having used translational research technology services. Over three-quarters of respondents reported that the NNE-CTR has been very or somewhat effective in building research capacity throughout northern New England (77%).

**Table 2.** Network and Northern New England Clinical and Translational Research (NNE-CTR) Experiences (*n* = 59)

Experiences with the Northern New England Community Engaged Research Network	
**Current engagement with the network** (*n* = 49)	
Participated in meetings	42 (86%)
Connected other community partners to the group	35 (71%)
Identified research priorities	33 (67%)
Advocated for a community voice	32 (65%)
Collaborated on a community health improvement project	31 (63%)
Identified partners to secure resources	28 (57%)
Assisted in dissemination of research	27 (55%)
Collected data for research	26 (53%)
Provided access to data for research	23 (47%)
**Network activities** (*n* = 48)	**Agree or strongly agree**
Increases opportunities for rural health research	41 (85%)
Values the community voice	40 (83%)
Exchanges ideas and knowledge	40 (83%)
Coordinates community-based research activities	39 (81%)
Respects the contributions of all partners	38 (79%)
Brings together diverse stakeholders	37 (77%)
Shares resources	37 (77%)
Develops new community-based research projects	36 (75%)
Promote opportunities to engage in joint research efforts	36 (75%)
Communicates a shared vision or goal	32 (67%)
Supports collective decision-making	32 (67%)
Meets regularly	27 (56%)
**Experiences with the NNE-CTR**	
**NNE-CTR services used** (*n* = 45)	
Community Engagement Research Navigation Services	28 (62%)
Professional Development Services	24 (53%)
Pilot Projects	20 (44%)
Research Navigation Services	19 (42%)
Translational Research Technology Services	6 (13%)
**To what extent have the services of NNE-CTR been effective in:** (*n* = 44)	**Very or somewhat effective**
Connecting researchers with community partners	33 (75%)
Building research capacity throughout northern New England	33 (75%)
Fostering meaningful dialogues between research teams and community experts	32 (73%)
Helping researchers design effective community-engaged research projects	31 (70%)
Implementing (or fostering) new joint research projects with the community	30 (68%)
Supporting community members through Community Engagement Research Navigation Services	30 (68%)
Improving my organizations’ capacity to conduct community-based research	26 (59%)

**Figure 1.**
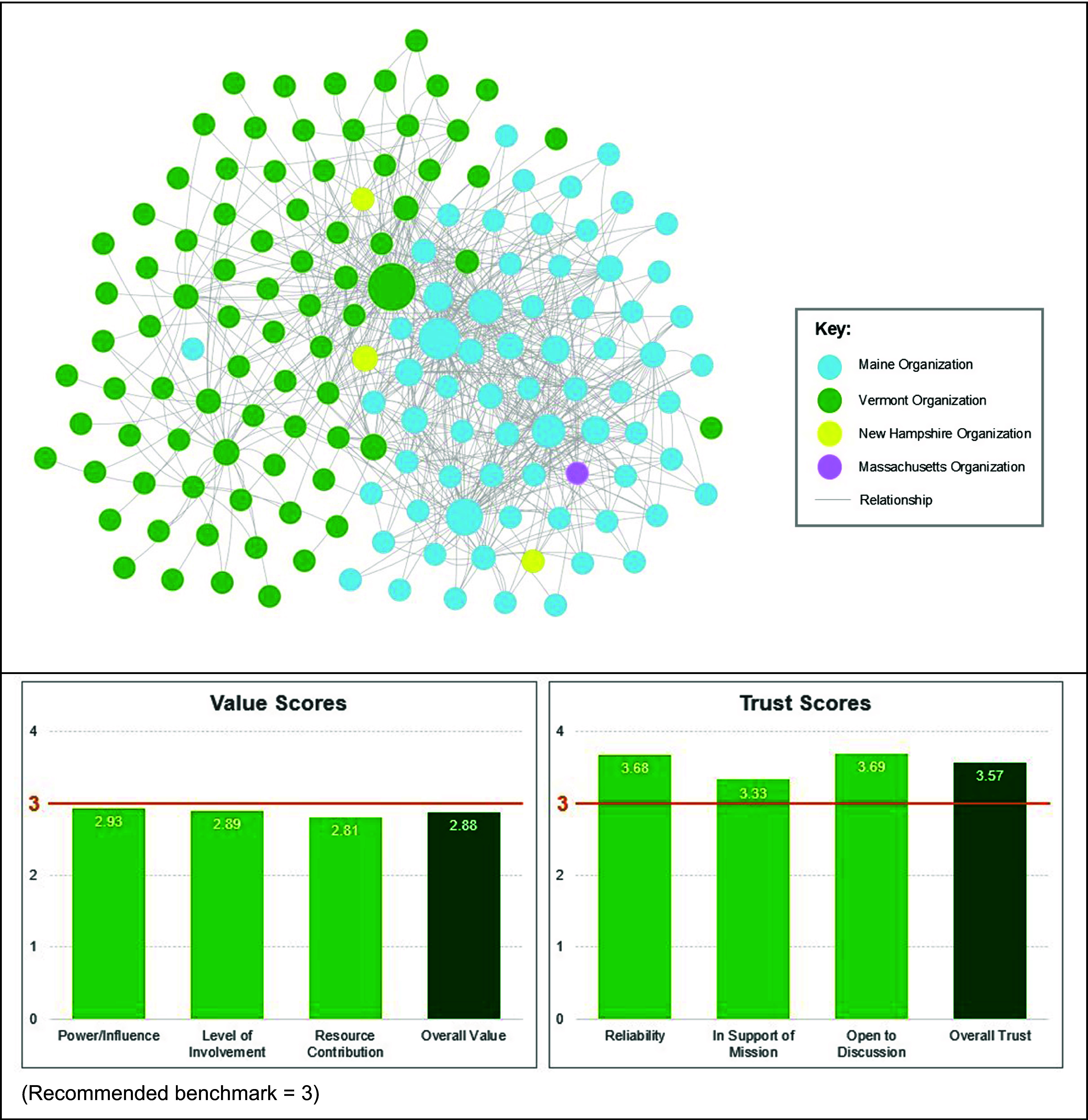
Northern New England Community Engaged Research Network Map and Trust/Value Scores.

Among the 160 organizations the 59 respondents could select as partners, 735 total partnerships were identified. As seen in Figure 1, the network map created in the PARTNER platform shows each organization as a circular node. The partnerships, or ties, identified by survey respondents are represented as lines between the nodes. The number of partners identified by individual respondents ranged from 0 to 71 (mean = 15.6, median = 13). Respondents then answered questions about each partner they identified. Most of these relationships were reported as long-standing, existing relationships (63%). About half of the relationships (51%) were categorized as cooperative or coordinated, which the PARTNER platform considers a sustainable level of intensity.

The validated relational metrics of value and trust each contained three indicators which are considered equally important. Value was measured by power and influence, level of involvement, and resource contribution. The NNE-Community Engaged Research Network’s scores for each of these value indicators were below the recommended benchmark of 3.00 for a healthy network, and the overall value score was 2.88. This score indicates a need to better leverage partners’ value within the network.

Trust among identified partners was measured by mission alignment, partner reliability, and openness to discussion. The NNE-Community Engaged Research Network’s scores for each of these trust indicators were above the recommended benchmark of 3.00, and the overall trust score was 3.57, indicating that a high level of trust among network partners already exists.

Respondents answered open-ended questions by describing perceived benefits of the NNE-Community Engaged Research Network. Themes from these responses centered around opportunities for connections, both to organizations and to research projects and expertise. Respondents also shared organizations to include as potential partners in future iterations of the survey.

## Discussion

Key findings from this evaluation included strong utilization of resources provided by the NNE-CTR and highly cooperative or coordinated relationships between partners. We also observed high levels of trust but more moderate reported perception of value in the engaged research network. The 2023 PARTNER survey results provided baseline data and visualizations to answer the evaluation team’s 4 research questions. As part of the overall evaluation plan, the survey will be repeated in 2025 and 2027. The goal is to increase the network’s reach and trust and value scores by 10% each time.

The PARTNER survey provides a variety of benefits to the CEO Core’s ongoing work to further strengthen community engagement, partnerships, bi-directional collaboration, and focus on community-identified research priorities. The results provided data to support perceptions of partnership strength, high levels of trust, and levels of engagement. These data allow CEO Core research navigators to focus on specific partners for additional outreach and/or communication.

Data related to elements of the value score provide specific topics of power/influence, level of involvement, and resource contribution for further exploration; this overall value score, less than the desired 3.0, provides a longitudinal focus for efforts in both Maine and Vermont to further strengthen types and intensity of interactions and community partner involvement. The CEO Core has previously observed a large power and cultural differential between academic and community-based organizations. However, progress is being made to engage network partners toward more collaborative engagement.

Research interests identified by respondents help CEO navigators in priority setting by connecting researchers in academic centers to community partners in Maine and Vermont in the identified focus areas. This information additionally supports and connects to other Cores. Overall, PARTNER survey data help the CEO Core build on community engagement and trust to align community and academic priorities, both short and long-term.

The flexibility of the PARTNER survey offered numerous benefits. The NNE-CTR evaluation team customized the modifiable survey items to answer its specific research questions. Through this process, the CEO Core recognized the opportunity to include additional questions unrelated to the research questions. While the CEO Core was excited to gain insights through social network analysis, they also had specific needs to learn more from community partners. Including those questions in the PARTNER survey was an efficient outreach strategy.

There are some important limitations to the survey results. While the trust and value scores offer insights into the strength of the NNE-Community Engaged Research Network, their validity would have been improved without the selection bias introduced by convenience sampling. When the survey is repeated in future years, the evaluation team will be able to examine how the sample of respondents varies and if there are differences in scores by subgroup. Additionally, the findings for the NNE-Community Engaged Research Network are limited to its network and not generalizable.

## Conclusions

As clinical and translational research networks increasingly prioritize community-engaged research efforts, using validated tools to measure and track relational attributes among organizations provides insights into relationships. The PARTNER platform is an accessible and adaptable tool that can meet this need.
